# Therapeutic management of neuro-oncologic patients - potential relevance of CSF liquid biopsy

**DOI:** 10.7150/thno.36884

**Published:** 2020-01-01

**Authors:** Louisa von Baumgarten, Jörg Kumbrink, Andreas Jung, Anna Reischer, Madeleine Flach, Sibylle Liebmann, Klaus H. Metzeler, Julian W. Holch, Maximilian Niyazi, Niklas Thon, Andreas Straube, Michael von Bergwelt-Baildon, Volker Heinemann, Thomas Kirchner, Christoph Benedikt Westphalen

**Affiliations:** 1 Department of Neurology, LMU Munich, Munich, Germany;; 2 Institute of Pathology, LMU Munich, Munich, Germany;; 3 German Cancer Consortium (DKTK), Heidelberg, Germany;; 4 Department of Medicine III, University Hospital, LMU Munich, Munich, Germany;; 5 Comprehensive Cancer Center Munich, University Hospital, LMU Munich, Munich, Germany;; 6 Department of Radiation Oncology, University Hospital, LMU Munich, Munich, Germany;; 7 Department of Neurosurgery, LMU Munich, Munich, Germany.

**Keywords:** Next Generation Sequencing (NGS), CNS Cancer, CSF, cfDNA, liquid biopsy.

## Abstract

**Background:** In the era of precision medicine, cancer treatment is increasingly tailored according to tumor-specific genomic alterations. The analysis of tumor-derived circulating nucleic acids in cerebrospinal fluid (CSF) by next generation sequencing (NGS) may facilitate precision medicine in the field of CNS cancer. We therefore evaluated whether NGS from CSF of neuro-oncologic patients reliably detects tumor-specific genomic alterations and whether this may help to guide the management of patients with CNS cancer in clinical practice.

**Patient and methods**: CSF samples from 27 patients with various primary and secondary CNS malignancies were collected and evaluated by NGS using a targeted, amplicon-based NGS-panel (Oncomine Focus Assay). All cases were discussed within the framework of a molecular tumor board at the Comprehensive Cancer Center Munich.

**Results**: NGS was technically successful in 23/27 patients (85%). Genomic alterations were detectable in 20/27 patients (74%), 11/27 (40%) of which were potentially actionable. After discussion in the MTB, a change of therapeutic management was recommended in 7/27 (26%) of the cases. However, due to rapid clinical progression, only 4/27 (15%) of the patients were treated according to the recommendation. In a subset of patients (6/27, 22%), a high number of mutations of unknown significance suggestive of a high tumor mutational burden (TMB) were detected.

**Conclusions:** NGS from cerebrospinal fluid is feasible in routine clinical practice and yields therapeutically relevant alterations in a large subset of patients. Integration of this approach into a precision cancer medicine program might help to improve therapeutic options for patients with CNS cancer.

## Introduction

Primary and secondary brain tumors still have a dismal prognosis. Due to the blood brain barrier, systemic chemotherapy is thought to have limited CNS penetration. Thus, therapeutic interventions often have to include surgical and radiotherapeutic approaches or, in the case of leptomeningeal tumor spread, the intrathecal delivery of cytotoxic agents^1^-^3^. Precision medicine and the use of targeted therapies, which are tailored to match individual tumor-driving mutations, have led to relevant changes of standard clinical care in patients with systemic cancer and have significantly improved outcomes for patients with different types of cancer such as melanoma^4^ and lung carcinoma 5.

Numerous clinical trials have shown that targeted therapies and immunotherapeutic agents can penetrate the blood-brain barrier and produce clinically meaningful intracranial response rates. Examples include EGFR and ALK inhibitors in non-small cell lung cancer, BRAF inhibitors in melanoma, HER2-targeting agents in breast cancer, and immune checkpoint inhibitors of CTLA4 or PDL1 in melanoma or non-small cell lung cancer 6-10. Therefore, the ability to analyze tumor genomics and monitor tumor evolution in patients with CNS malignancies has the potential to improve outcomes in patients with CNS cancer as well.

So far, molecular information of CNS cancer is usually obtained from tissue samples harvested by open resection or minimally invasive stereotactic biopsies. These invasive procedures may bear a considerable risk (e.g. due to tumor location in highly eloquent locations). Furthermore, repetitive tumor sampling over time may not be feasible.

An alternative is NGS (next generation sequencing) based analysis of cell-free total nucleic acids (cfTNA) in “liquid biopsies”. cfTNA comprises nucleic acid (NA) fragments (DNA and RNA) released from cells into the peripheral blood during apoptosis and necrosis. Within the cfTNA a small fraction of tumor-derived cfTNA, called circulating tumor TNA (ctTNA), shed from tumor cells is present, which can be analyzed for genetic alterations.

“Liquid biopsy” or the mutational analysis of ctTNA in accessible body fluids by targeted next generation sequencing (NGS) is commonly applied in patients with systemic cancer and the resulting comprehensive genomic information can give patients access to targeted therapies 11. ctTNA can also be detected in the peripheral blood of a fraction of patients with primary and secondary CNS malignancies^12^, ^13^. However, its detection rate is relatively low compared to that in patients with systemic cancer 14-17. The paucity of ctTNA in the peripheral circulation in CNS cancer is most likely related to the blood-brain barrier, which prevents the efflux of cfTNA from the CNS. Therefore, CSF is a potentially attractive sampling source. Several smaller studies collected CSF and identified cfDNA of primary and metastatic brain tumors using different techniques 18-20. cfTNA could be detected in an NGS approach in the CSF of up to 50-74% of the patients with primary brain tumors^18^, ^21^ and in 63% of the patients with CNS metastasis, but not in patients without CNS involvement by cancer^19^. In line with this, peripheral ctDNA levels in patients with cerebral metastasis of solid tumors were lower than those detected in the CSF, except for patients with significant systemic disease burden. Furthermore, CSF cfTNA better represents the genomic alterations found in brain metastasis than plasma ctTNA^20^. Therefore, in the context of CNS-related cancers, cfDNA analysis from CSF may represent a powerful tool leading to more promising diagnostic yield than from peripheral blood.

In our study, we investigated the feasibility of analyzing the ctDNA and ctRNA within the CSF of patients with CNS cancer. Therefore, an amplicon-based NGS assay was applied that targets not only point mutations and deletions but also therapeutically relevant amplifications and gene fusions to facilitate access to targeted therapies within the framework of an interdisciplinary molecular tumor board.

## Material and Methods

### CSF collection and sample processing

Between December 2016 and May 2018, we collected CSF samples from 27 patients with cancer who underwent lumbar puncture as part of their routine clinical management. All patients signed informed consent for the analysis of their clinical data within a broad prospective registry conducted at Clinical Cancer Center of the Ludwig Maximilian University Munich (“The informative patient”), which was approved by the local ethics committee according to the Declaration of Helsinki. All patients were discussed in an interdisciplinary tumor board before and after molecular testing, had a clinical indication for molecular testing, and were informed about the purpose of the molecular analysis by the treating physician. CSF samples were collected in 10-ml Cell-Free DNA BCT blood collection tubes (Streck, La Vista, NE, USA) to prevent the release of genomic DNA from cells within the sample and the absorption of cell-free (CF) nucleic acids (NA).

### Cell-free nucleic acid isolation

CSF nucleic acids were isolated from 1.5-6 ml CSF (median 3 ml) using the QIAmp Circulating Nucleic Acid Kit (Qiagen, Valencia, CA, USA) according to manufacturer's instructions and quantified with a Qubit 3^TM^ Fluorometer (Thermo Fisher Scientific, Waltham, MA, USA).

### Panel sequencing

Genomic profiling of samples by targeted NGS was performed by using a commercially available assay (Oncomine™ Focus Assay, Thermo Fisher Scientific) covering 30 kb of coding DNA on an Ion Torrent Personal Genome Machine^TM^ (PGM), as described previously^22^. Briefly, the Oncomine^TM^ Focus Assay (OFA) is a multi-biomarker NGS system that enables the detection of variants in 52 key solid tumor genes (see Supplementary Table 1). These genes are well-characterized in the published literature and can partly be therapeutically addressed by targeted FDA approved agents, part of National Comprehensive Cancer Network (NCCN) guidelines, or in clinical trials. The OFA allows concurrent analysis of DNA and RNA to simultaneously detect single nucleotide variants (SNVs) and insertions/deletions (indels) in mutation hotspots as well as copy number variations (CNVs) and gene fusions 22.

cDNA synthesis, library preparation, equalizer PCR, usage of the IonChef™ pipetting station, and Ion PGM^TM^ Chip loading was performed according to the manufacturer's instructions. Briefly, the Invitrogen™ SuperScript™ VILO™ cDNA Synthesis Kit (Thermo Fisher Scientific) was used for reverse transcription prior to library preparation for the RNA panel. 20 ng DNA and 100 ng RNA were used for library generation utilizing the OFA, Select Library kit (Thermo Fisher Scientific). The Ion AmpliSeq™ Sample ID Panel (Thermo Fisher Scientific) was added to the library-generating PCR reaction for proper sample identification. The libraries were equalized with the Ion Library Equalizer™ Kit. Next, a maximum of 6 libraries (6 DNA and 6 RNA) were transferred to the IonChef™ pipetting station for library enrichment and subsequent Ion-318™ Chip loading (Ion PGM™ Hi-Q™ View Chef Kit, Thermo Fisher Scientific). Prepared chips were loaded into the IonTorrent PGM™.

Data analysis was performed with the dedicated software provided by the manufacturer, the Integrated Genomics Viewer (IGV, Broad Institute), and a proprietary database calling tool for the identification of single nucleotide polymorphisms (SNPs) and the tumor genetic evaluation of the identified alterations (Supplementary Fig. S1). Sequencing data were aligned to the human reference genome hg19 using Torrent Suite™ (ver. 5.2). Analysis, filtering, and annotation of variants were carried out with Ion Reporter™ (ver. 5.2). An automatic workflow (Oncomine Focus v2.1) with preconfigured parameter settings (Oncomine Variants 3% CI SCNV ploidy ≥ gain of 2 over normal) was used. The following sequencing quality metrics were used to determine the success of the analysis: 1) DNA: average base coverage depth ≥1000; 2) DNA: amplicons having at least 100 reads: ≥90%; 3) RNA (gene fusions): total Mapped Fusion Panel Reads ≥10000 4) RNA: all 5 expression controls were expressed. DNA alterations with a total coverage ≥200 reads and allelic frequencies >3% or gene fusions detected by RNA analysis ≥20 reads were considered positive. All candidate mutations were further reviewed using the IGV.

One sample (#6, Ewing sarcoma) was additionally analyzed with the Archer® FusionPlex® Sarcoma system (ArcherDX, Inc., Boulder, CO, USA) on an Ion Torrent PGM^TM^ platform for sarcoma-specific gene fusions not present in the OFA panel. The Archer Sarcoma kit is a targeted sequencing assay to simultaneously detect and identify fusions of 26 genes associated with soft tissue cancers (Supplementary Table 1). The library preparation was executed as described in the vendor's manual using 250 ng RNA. The final library concentration was measured with an Experion^TM^ bioanalyzer (Biorad, Hercules, CA, USA). The analysis was performed with the Archer Analysis 5.1 site using Archer Comprehensive Targets v1.1, FusionPlex Sarcoma AK0032 v1.0 and the default quality metrics recommended by the manufacturer. CSF of three lung cancer patients (#1, #4 and #27) whose cancer progressed under tyrosine kinase inhibitor (TKI) treatment were analyzed for TKI resistance mutations in the EGFR gene (T790M mutation). Therefore, the smaller AmpliSeq Colon and Lung Cancer v2 DNA only panel (Thermo Fisher Scientific) containing 22 NSCLC related oncogenes and tumor suppressor genes (*AKT1, ALK, BRAF, CTNNB1, DDR2, EGFR, ERBB2, ERBB4, FBX7, FGFR1, FGFR2, FGFR3, KRAS, MAP2K1, MET, NOTCH1, NRAS, PIK3CA, PTEN, SMAD4, STK11, TP53*) was used in this situation.

### Potential actionability and molecular tumor board

An actionable alteration was defined as a characterized alteration that was either the direct target or a pathway component that could potentially be targeted by at least one FDA- and/or EMA-approved drug (in the same or another indication) or by an investigational drug in a clinical trial. Potential actionability was crosschecked by at least two investigators and mining of literature of actionable mutations and their clinical relevance was performed for each case (KHM, CBW, AJ, TK). All patients were discussed, and individual therapeutic recommendations were made within the framework of an interdisciplinary molecular tumor board, which is part of the “Molecular Diagnostics and Therapy” program at the Clinical Cancer Center of the Ludwig Maximilian University Munich.

### Data extraction and analysis

Demographic information, such as gender and age, as well as the clinical symptoms, laboratory testing, and results of neuroimaging, if available, were analyzed by review of the electronic medical chart. Furthermore, dates of sample collection, test results, list of actionable alterations data, and the result of the molecular tumor board were analyzed.

### Data availability

Anonymized data will be shared on request from any qualified investigator.

## Results

### Patient characteristics

Overall, we included 27 patients in our analysis (Table 1). The median age was 58 years (range 22-82 years) and 16 patients were female. 23 patients had brain metastases from solid tumors. The most common primary tumors were breast (10/27, 37%) and non-small cell lung cancer (NSCLC) (8/27, 30%). Other tumor entities included colorectal cancer (CRC, 1/27), Ewing sarcoma (1/27), cholangiocellular carcinoma (CCC, 1/27), melanoma (1/27), and gastric cancer (1/27). Four patients suffered from primary brain tumors like glioblastoma (GBM, 2/27) and primary CNS lymphoma (PCNSL, 2/27).

Cytology revealed malignant cells in the CSF of 52% of the patients (14/27). MRI of the brain was indicative of leptomeningeal metastasis (LM) in 59% (16/27) and MRI of the spine in 44% (12/27) of the patients. Overall, 70% (19/27) of the patients had CSF and/or MRI positive for LM. 81% of the patients (22/27) had radiographic evidence for a parenchymal tumor manifestation in the brain or the spine**.**

### Sequencing metrics

We isolated nucleic acids from 1.5-6 ml CSF (median: 3 ml). cfDNA and cfRNA could be isolated in all cases. cfDNA was used for the analysis of point mutations, insertions/deletions, and copy number variations. cfRNA was applied for the detection of gene fusions. NGS was successful in 23/27 (85.2%) of the patients. In 4 patients with low cfDNA content (median 0.5 ng/µl, range 0.4-2.5 ng/µl), the sequencing metrics (target base coverage at 100 reads: median: 75%, range: 71-78%) did not meet our quality standards and were excluded due to our strict quality thresholds. Sequencing metrics are summarized in Table 2 and Table 3.

### Actionable genomic alterations detected in the cfTNA of CSF samples

In our patient cohort, somatic alterations were detected in 74% of the patients (20/27). Among patients with somatic alterations, we observed a median number of 3 (range 1-47) mutations in the Oncomine panel. 40% (11/27) of the patients carried potentially clinically actionable alterations which can principally be targeted by drugs already approved or currently in clinical trials. We found targetable alterations in the *EGFR, BRAF, NTRK1, PIK3CA, MET, ROS1,* and *MTOR* genes. cfTNA with somatic mutations was detected in 62% (5/8) of the patients with CNS cancer who had negative findings for LM in CSF cytology and MRI (Table 4).

### Therapeutic recommendation of the molecular tumor board (MTB) and clinical patient management

**Figure 2:**
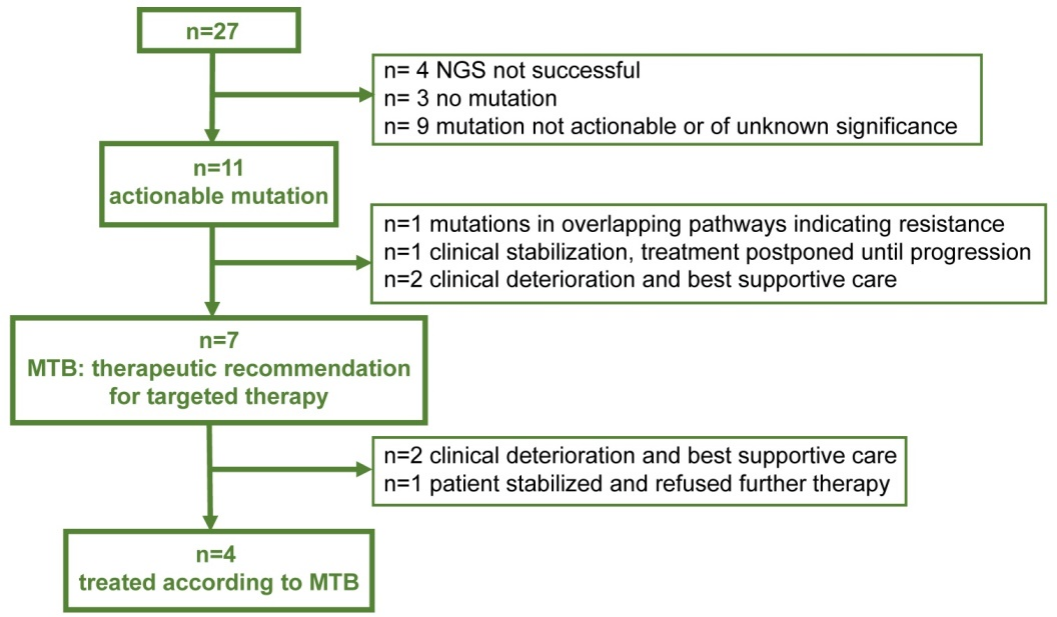
** Therapeutic management** Flow Chart indicating the yield of actionable mutations in 27 patients, respective recommendations of the Molecular Tumor Board (MTB) and actual treatment

Although 11/27 (40%) CSF-samples yielded potentially clinically actionable alterations, a change in the therapeutic management was recommended by the interdisciplinary MTB in 7/27 (26%) patients (Figure 2 and Table 4). In 4/27 patients (#22, #24, #25, #27) no targeted therapy was recommended although an actionable mutation was discovered: In one patient, several mutations were identified in overlapping pathways, potentially indicating resistance to respective targeted approaches (#22). In another patient (#24), tumor control and clinical stabilization after whole brain radiotherapy (WBRT) was achieved and an initially suspected LM could be ruled out. Therefore, targeted therapy was postponed until progression. One patient showed rapid and unforeseen clinical deterioration (#25) and, due to the relatively low allele frequency of the alterations found, the clinical benefit of a possible targeted approach for the tumor entity was deemed unlikely. One patient (#27) progressed on afatinib, suggesting an EGFR resistance mutation. NGS from CSF revealed the previously detected primary EGFR-mutation; however, neither the targetable T790M resistance mutation nor another relevant actionable mutation was detected. Furthermore, the patient showed rapid clinical deterioration and therefore received best supportive care.

In 7/27 patients with a recommended change of the therapeutic regimen, however only 4/27 (15%) of the patients could be treated according to the recommendations, as two patients showed unexpected clinical deterioration (#3, #7) and were treated with best supportive care and one patient (#17) refused further therapy.

In the following paragraph, we briefly summarize the therapeutic recommendations of the molecular tumor board.

A 45-year-old male patient was referred to our hospital with headache and progressive cranial nerve palsy. Three weeks earlier, the patient had been diagnosed with non-small cell lung cancer (NSCLC) (adenocarcinoma of the lung, UICC stage IV, cT1, cN2, cM1b) with lymph node and bone metastases by tissue biopsy in an external hospital. Based on this diagnosis, one cycle of palliative platinum-based chemotherapy was initiated by the external physicians. Information on the molecular profile of the tumor was lacking. Lumbar puncture and MRI of brain and spine revealed LM and targeted NGS sequencing from the CSF identified an activating EGFR mutation (Exon 21) (see also #1, Table 4), which was later confirmed in the primary tumor biopsy by PCR as well. Systemic chemotherapy was discontinued and the treatment regimen was changed to erlotinib p.o. and intrathecal methotrexate. Under treatment, MRI revealed regression of the adherent LM. Cranial nerve palsy subsided. The systemic tumor load and non-adherent LM had been stable for 25 months when this manuscript was written (Figure 2).

Similarly, in a 58-year-old female patient (#4, Table 4) with newly diagnosed NSCLC (adenocarcinoma, UICC stage IV, cT2b, cN0, cM1c) with pulmonary, bone, brain, and leptomeningeal metastasis, systemic chemotherapy was switched to *in-label* afatinib in addition to intrathecal methotrexate injections after activating EGFR mutations (Exon 18, p.G719C; exon 20, p.S768I) were diagnosed by liquid CSF biopsy. Furthermore, she received whole brain radiotherapy (WBRT, 30 Gy, 3 Gy per fraction) due to a high cerebral metastatic burden. Further analysis revealed that the activating EGFR mutations were confirmed in the bone metastasis. However, the primary tumor did not contain EGFR exon 18 and 20 mutations but an activating EGFR exon 19 mutation (p.P753Q) which was observed in neither the CSF liquid biopsy nor in the bone metastasis. Under therapy, the patient showed a partial systemic and intracranial response for 5 months. Then she underwent stereotactic re-irradiation due to isolated intracranial progression. The systemic tumor load was stable and afatinib was continued. Eleven months after diagnosis, she showed a rapid systemic progression. After one cycle of pemetrexed the therapy was not continued due to her clinical deterioration and she succumbed to her disease under best supportive care 12 months after the initial diagnosis.

A 66-year-old male patient with systemically controlled NSCLC was diagnosed with two progressing cortical cerebral metastases after WBRT (#15). NGS from CSF revealed an *EZR/ROS1* fusion as well as an *MTOR* mutation. Due to the *EZR/ROS1* fusion, an *in-label* therapy with a CNS-penetrating ROS-inhibitor (ceritinib, crizotinib) was recommended by the molecular tumor board. At the time of manuscript preparation the patient had received crizotinib for 6 months and had stable systemic and cerebral disease.

A 62-year-old female patient with systemically controlled, metastasized (bone and lymph node), hormone receptor positive breast carcinoma was diagnosed with new cerebral metastases and adherent as well as non-adherent LM (#23). NGS from CSF revealed an amplification of the *FGFR1* gene as well as an amplification of the *MYC* gene.

Dysregulation of FGFR signaling can lead to downstream activation of mitogen activated protein kinase (MAPK) and phosphoinositide-3-kinase (PI3K)/AKT pathways 23 and it has been shown, that patients with FGFR amplifications profit from PIK3CA/AKT directed therapy with everolimus 24. Furthermore, FGFR amplification may confer resistance to CDK4/6 inhibitors 25.

The molecular tumor board recommended an *in-label* therapy with everolimus in addition to systemic exemestane. Furthermore, the patient received WBRT (30 Gy) and intrathecal injections of methotrexate until CSF was cleared of atypical cells. Under the subsequent treatment with everolimus and exemestane the patient had been stable for 6 months when this manuscript was written.

However, three patients did not receive the treatment that was recommended by the molecular tumor board (Figure 2).

In a 71-year-old male patient with peritoneal and abdominal metastasis of a cholangiocellular carcinoma (CCC) MRI revealed adherent spinal and cranial leptomeningeal metastasis. NGS from CSF detected a targetable *BRAF* mutation (p.V600E) as well as an activating *ERBB2*-mutation (#3, Table 4). The current literature (one basket study including one patient with CCC 26 as well as different case reports 27-29) corroborates the efficacy of BRAF-inhibiting monotherapy with vemurafenib and a combined BRAF- and MEK-inhibition in patients with CCC. In view of the additional *ERBB2* mutation, which affects the RAS/RAF/MEK/ERK pathway 30, the tumor board recommended an off-label therapy with combined BRAF- and MEK-inhibition together with the intrathecal application of methotrexate. However, due to a rapid clinical deterioration, the patient refused further therapy and best supportive care was initiated.

A 69-year-old female patient with bilateral hormone receptor positive breast carcinoma with bone metastasis of the skull and cutaneous tumor infiltration was diagnosed with non-adherent and adherent LM and novel brain metastasis (#7, Table 4). WBRI was refused by the patient but she was willing to undergo chemotherapy. NGS from CSF revealed a *TPM3-NTRK1* fusion. Patients with *NTRK-*rearranged tumors have achieved robust and durable responses to treatment with TRK inhibitors in clinical trials 11, 30. Therefore, in addition to the intrathecal application of methotrexate, the molecular tumor board recommended treatment with entrectinib, preferentially within a clinical trial (NCT02568267: Basket Study of Entrectinib (RXDX-101) for the treatment of patients with solid tumors harboring *NTRK1/2/3, ROS1* or *ALK* Rearrangements (Fusions), STARTRK-2). However, due to rapid clinical deterioration, the patient could not be included in the clinical trial.

A 67-year-old female patient (#17 Table 4) with breast carcinoma was referred to our clinic with headache and acute, progressive paraparesis. CSF cytology was negative for atypical cells, but a *PIK3CA* mutation was found in the NGS analysis. MRI of the spine revealed contrast enhancement of the caudal spinal cord. cMRI was not possible due to a cochlea implant. The molecular tumor board recommended a therapy with everolimus. However, repetitive CSF punctures over the course of 8 weeks did not confirm LM and the symptoms did not progress further but instead improved slightly. Therefore, therapy was not initiated by the treating physician, which was in accordance with the patient's refusal to undergo further therapy. The patient was re-evaluated twice over a 9-month period without any evidence of clinical or radiological (MRI of the spine) progression.

Besides known actionable mutations somatic alterations with unclear therapeutical/clinical significance (VUS, variant of unknown significance) were discovered in 22% (6/27) of the patients (Supplementary table 3). A high number of somatic alterations/VUS were found in 3 patients with breast carcinoma (#5, #9, #17), 2 patients with cerebral lymphoma (#16, #21), and 1 patient with melanoma (#22) (see Table 4 and Supplementary Table 3). The number of somatic alterations in these cases ranged from 7 to 47 (median: 11 variants). This increased number of mutations in comparison to other cases suggests a high tumor mutational burden (TMB high) which has been associated in recent studies with response to immune checkpoint inhibition in various cancers^12^, ^31^, ^32^. The quantification of TMB requires a sequencing assay that covers a territory of at least 0.8 megabases (Mb) ,while the Oncomine Focus assay covers only about 0.03 Mb and is thus not applicable for TMB measurements. Therefore, further diagnostic workup with a suitable panel to confirm TMB^high^ was recommended by the molecular tumor board.

## Discussion

Our study demonstrates the feasibility of detecting clinically relevant somatic alterations in the CSF of patients with CNS cancer. Importantly, this study was conducted using an amplicon-based, commercially available NGS panel. We show here, that CSF samples from patients with and without leptomeningeal involvement of CNS cancer contain cfTNA and that a targeted standardized amplicon-based NGS assay generates clinically relevant data. All patients were discussed in an interdisciplinary molecular tumor board and targeted therapy was recommended with respect to the individual clinical history and molecular results obtained by NGS. A therapeutic recommendation was made for more than one quarter of all patients. However, due to rapid clinical deterioration and patient refusal, only 15% (4/27) actually received the therapy recommended by the molecular tumor board. This shows that leptomeningeal disease still has a poor prognosis and that patients' samples are often subjected to extend molecular testing at rather late stages of their disease. It has been suggested that patients with late-stage disease often have only limited benefit from precision cancer medicine 33.

In our studies, it was possible to successfully analyze samples with a low content of CSF nucleic acids using both the DNA and RNA panel of a commercially available NGS solution. This is in line with previous publications that demonstrate the ability to sequence low concentrations of cfDNA in the CSF 18-21, 34-36. So far, the fraction of tumor-derived DNA has been considered to be higher in the CSF than in the plasma due to the relative absence of non-tumor derived DNA, thus offering the possibility to detect somatic alterations with low allelic frequencies^18^, ^20^.

We identified somatic alterations in 74% of the (20/27) patients with a median of 3 (range 1-47) mutations per case. However, 11% of the samples (3/27) did not show any detectable alteration. In these cases we cannot exclude the possibility that 1) genomic alterations are not covered by the small NGS panel used (~30 kb) and thus were not detectable and 2) that, although a high average coverage of approximately 15000 reads was achieved, the sensitivity was still too low to detect the varying and usually low amounts of ctTNA within the cfTNA (“needle in a haystack”). However, compared to larger targeted NGS assays or whole genome sequencing, we were able to increase the coverage depth and thus sensitivity by 5 to 10 fold in comparison to our routine tissue analyses. Furthermore, it represents a cost-effective solution for clinical practice 22. In addition, cfTNA levels may be limiting the capacity to perform more extensive NGS testing in the CSF in some patients.

We identified targetable alterations in 40% (11/27) of the patients. However, after discussion in our interdisciplinary molecular tumor board, therapeutic recommendations could only be provided for 26% (7/27) of the patients. This highlights the fact that personalized medicine, relying on the genetic characterization of CNS malignancies, with its inherent potential pitfalls and difficulties in interpretation should be pursued within the framework of an interdisciplinary molecular tumor board to avoid unnecessary and potential harmful therapeutic interventions.

In one breast cancer patient with radiologically and clinically suspected LM, cfDNA showed a targetable *PIK3CA* mutation. However, no treatment was initiated due to clinical stabilization and patient refusal. For 11 months, the patient showed no clinical or radiological progression and sequential CSF punctures were negative for malignant cells. Overall, LM seems unlikely and it cannot be ruled out that the positive NGS result reflects blood derived cfDNA. However, unfortunately no matching blood sample was analyzed to support this hypothesis.

In this study we found a high number of somatic alterations (median 11, range 7-47 in 30 kB coding DNA screened) in a significant fraction of CSF samples (22%, 6/27). These high numbers of mutations point to an increased tumor mutational burden (TMB). TMB is quantified as a number of somatic mutations within an exonic territory of at least 0.8 Mb as mutations per megabase (mut/Mb). This requirement is a potential drawback of the NGS panel used in this study as it covers only 0.03 Mb and thus cannot be utilized for the quantification of TMB. However, determining TMB from liquid biopsies (blood/liquor-based TMB) is very expensive because 1) a huge territory needs to be sequenced 2) at a high coverage to overcome the sensitivity problem (ctDNA accounts usually for less than 5% of the total cfDNA 31).Therefore, liquid-based TMB analysis cannot be performed on smaller sequencing machines such as the Ion PGM used in this study.

TMB metrics can be used to classify tumors into classes with low, medium, and high mutational burden. A high TMB (defined according to various cut-offs ranging from ≥10 to ≥20 mutations per Mb) might increase the number of distinct neoepitopes presented on the surface of tumor cells and hence lead to greater tumor immunogenicity. It has been validated as a predictive biomarker of efficacy of checkpoint inhibitors in several tumor types 27, 32. TMB was initially calculated using whole-exome sequencing (WES) of up to 60 Mb of the genome. Alternatively, sequencing of a reduced set of relevant genes with a higher sensitivity has been validated to determine TMB 27. The fact that we found a significant fraction of samples with a comparatively high amount of genomic alterations is an argument, especially in terms of a potential therapeutic alternative, to recommend TMB measurement with a validated TMB panel.

Our study has other limitations. We analyzed a comparatively small patient cohort with multiple different types of CNS cancer, and no long-term follow up is available to determine the actual clinical benefit for the majority of patients. Although our results show that NGS from CSF can help to guide therapeutic recommendations, further larger prospective studies are needed to clarify whether individualized targeted therapeutic approaches based on CSF NGS results indeed translate into a clinical benefit for the patient.

Furthermore, it would be of great interest to systematically compare the sequencing results of CSF, blood, brain parenchymal metastasis, and primary cancers, which was beyond the scope of our study.

Cancer cells continuously acquire new mutations due to genomic instability and/or selective pressure from the tissue microenvironment and clinical treatment. Recent data indicate that CNS manifestations of systemic cancer often carry genetic alterations that can differ from those observed in primary tumors and systemic metastasis 19, 37-40. In this respect, CNS metastases seem to harbor more clinically actionable mutations than the primary tumor and might thus better respond to targeted therapies. In a series of 86 paired cases of primary tumors and brain metastases, 53% of the brain metastases had one or more actionable mutation not found in the primary tumor 38. Similarly, CSF cfDNA was shown to harbor drug resistance mutations not present in the primary tumor 19. This heterogeneity is very relevant, as the majority of patients with CNS metastasis do not undergo surgery or biopsy for brain metastases and therefore, potential clinically actionable mutations or mechanisms of resistance in the CNS might not be identified. However, especially for primary brain tumors, cfDNA analysis from the blood may represent a less invasive alternative to CSF analysis requiring a lumbar puncture, which, under some circumstances (elevated intracranial pressure) may even be contraindicated. It will therefore be relevant to evaluate how far cfDNA analysis from the blood in this situation qualitatively represents the brain tumor lesion.

Furthermore, several studies have pointed out, that the absolute amount of cfDNA in the CSF might represent a predictor for therapeutic response 20, 26, 34. Furthermore, serial analysis of cfDNA in the CSF by NGS can unravel potential resistance mechanisms towards targeted therapies 19.

In the future, it will therefore be relevant to further evaluate the utility of cfDNA analysis as marker of therapeutic success. For this purpose, larger prospective studies need to be conducted enrolling patients to monitor cfDNA samples from CSF prior to, repeatedly during, and after therapeutic interventions.

In conclusion, our study highlights that the collection and genomic profiling of CSF using a commercially available, amplicon-based NGS approach is technically feasible and identifies targetable genetic alterations in a substantial subset of patients. In the framework of a molecular tumor board it proved helpful to identify patients for novel targeted therapeutic approaches in a real-life scenario. Future larger prospective trials should solidify the clinical benefit of patients with CNS cancer receiving targeted therapies according to NGS-based CSF analysis. Additionally, the value of genomic profiling as a marker of clinical response to therapy, analogous to plasma cfDNA, should be analyzed.

## Supplementary Material

Supplementary figures and tables.

## Figures and Tables

**Figure 1:**
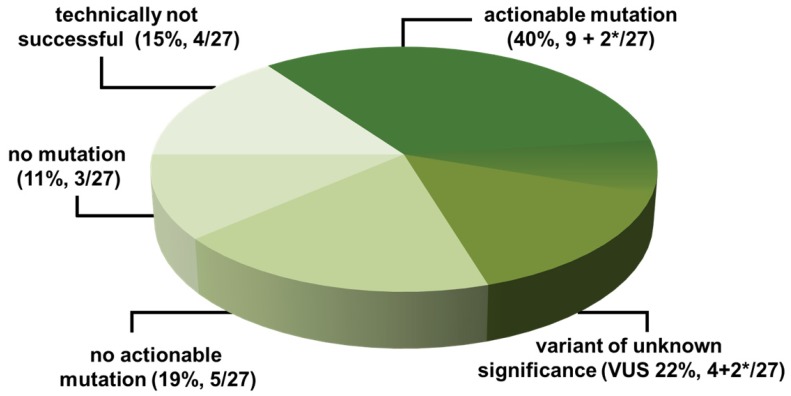
** NGS-Results** NGS-Analysis from CSF is technically feasible and yields tumor genetic mutations in the majority of patients analyzed. VUS: variants of unknown significance, *2 patients had both: actionable mutations and VUS.

**Figure 3:**
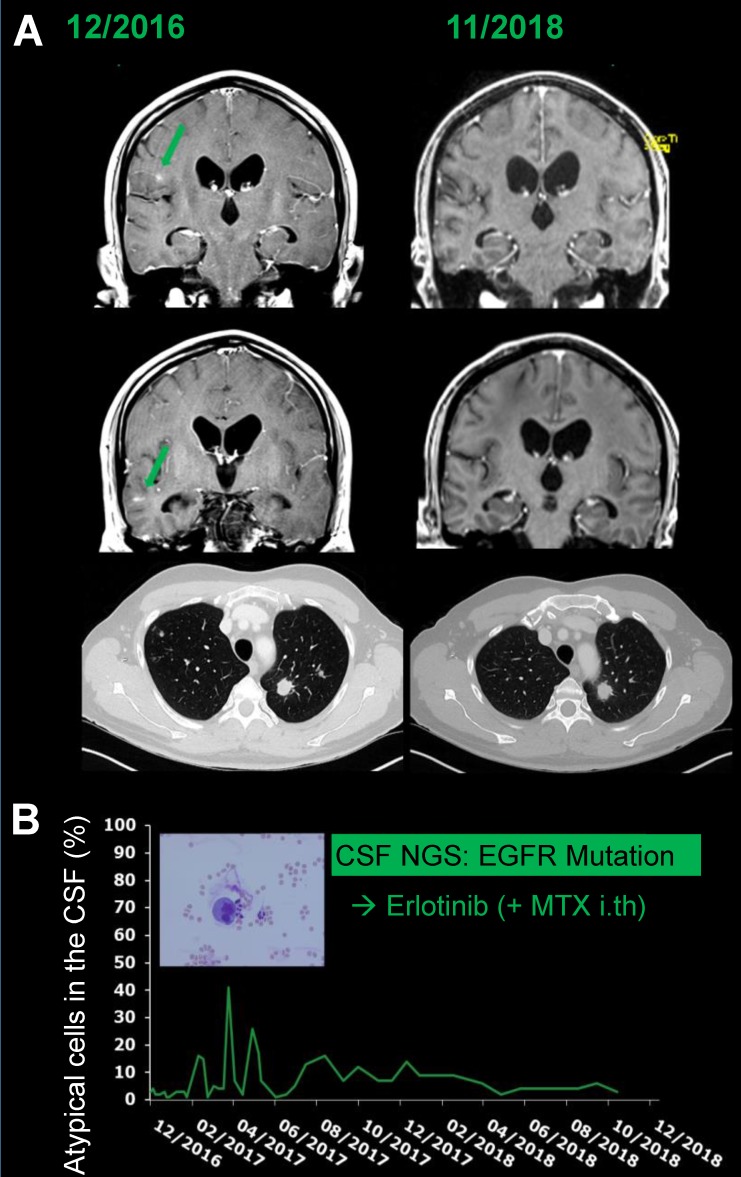
** Illustrative patient example** 45-year-old male patient (#1) with newly diagnosed metastatic (leptomeningeal, bone, lymph nodes, pulmonary) NSCLC with actionable mutation (EGFR Exon 21) diagnosed by NGS from CSF. **A**: Note the exceptional clinical course under treatment with oral erlotinib and intrathecal MTX with regression of preexisting contrast enhancing metastatic lesions (green arrows) and stable pulmonary disease over 24 months. **B:** Non-adherent LM was diagnosed by cytology and could not be eradicated; however, the percentage of atypical cells in the CSF remained low for the whole treatment period.

**Table 1:** Patient characteristics.

Median age (range)	58 (22-82)
**Gender women %, (N)**	59 (16/27)
**Secondary brain tumors %, (N)**	85 (23/27)
Breast	37 (10/27)
Lung	30 (8/27)
others	19 (5/27)
**Primary brain tumors %, (N)**	15 (4/27)
GBM	7 (2/27)
PCNSL	7 (2/27)
**CSF cytology %, (N)**	
Positive	52 (14/27)
Negative	48 (13/27)
**MRI brain %, (N)**	
Positive for LM	59(16/27)
Negative for LM	41(11/27)
Not performed	4 (1/27)
MRI spine %, (N)	
Positive for LM	44 (12/27)
Negative for LM	41 (5/27)
Not performed	37 (10/27)
**MRI and/or CSF %, (N)**	
Positive for LM	70 (19/27)
Negative for LM	30 (8/27)
**Parenchymal tumor manifestation %, (N)**	
Positive	81 (22/27)
Negative	19 (5/27)

**Table 2:** RNA Sequencing metrics.

RNA Sequencing	Total/avg	Threshold	Minimum observed*	Maximum observed*
RNA isolated (ng/µl)	4.21 (median: 1)	na	0.3	40.6
Samples tested (n)	24	na	na	na
Successfully analyzed (%)	83.33	na	na	na
Mapped fusion reads (n)	282742.50	10000	42322	832118
Fusion reads (n)	4	na	0	1

na, not applicable. * per patient sample

**Table 3:** DNA Sequencing metrics.

DNA Sequencing	Total/avg	Threshold	Minimum observed*	Maximum observed*
DNA isolated (ng/µl)	8.44 (median: 1)	na	0.1	110
Samples tested (n)	27	na	na	na
Successfully analyzed (%)	85.2	na	na	na
Average base coverage depth	14679.30	4000	4167	20717
Target base coverage at 100x (%)	98.78	90	92.87	100
Uniformity of coverage (%)	89.74	70	74.67	99.99
Mutation reads (n)	112	na	0	40
Mutation allele frequency (%)	na	3	3	66.55
Amplification detection (n)	3	na	0	2

na, not applicable.* per patient sample

**Table 4:** NGS results and clinical significance.

#	Tumor	LM	PM	Gene	Alteration	Exon	AF (%)	Recommendation
1	NSCLC	+	-	EGFR	c.2573T>G,p.Leu858Arg	21	8.7	Erlotinib
2	NSCLC	+	+	JAK3	c.1477A>T, p.Ser493Cys	11	8.0	pStat3 testing (if positive Tocatinib)
3	CCC	+	+	BRAF	c.1799T>A p.Val600Glu	15	15.8	Combinded BRAF- and MEK- nhibition
				ERBB2	c.2033G>A p.Arg678Gln	17	5.5	
4	NSCLC	+	+	EGFR	c.2155G>T, p.Gly719Cys	18	64.1	Afatinib
				EGFR	c.2303G>T, p.Ser768Ile	20	66.6	
5	Breast	+	+	ERG4	TMPRSS2(1) - ERG4(4) fusion, chr21:42880007 - chr21:39817544 *Probably TMB high			TMB testing, if TMB high checkpoint inhibition
6	Ewing Sarcoma	+	+	FUS	FUS(16) - DHX57(2) fusion, chr16: 31196264, chr2:39095397			not actionable
7	Breast	+	+	NTRK1	TPM3(7) - NTRK1(10) fusion, chr1:154142875 - chr1:156844362			Screening for clinical trial (Entrectinib, XDX-101)
8	Breast	+	-		no mutation			not actionable
9	Breast	+	+	AR	c.2630T>C, p.Phe877Ser	8	7.6	TMB testing, if TMB high checkpoint inhibition
				CTNNB1	c97T>C; pSer33Pro Probably TMB high	3	6.9	
10	CRC	+	+		not successful			
11	Breast	+	+		no mutation			not actionable
12	GBM	+	+		no mutation			not actionable
13	NSCLC	-	+		not successful			
14	GBM	-	+		no mutation			not actionable
15	NSCLC	-	+	ROS1	EZR(10) - ROS1(34) fusion, chr6:159191795 - chr6:117645578			Crizotinib or Ceritinib
				MTOR	c.6604T>C, p.Phe2202Leu	47	7.7	
16	PCNSL	-	+		*Probably TMB high			TMB testing, if TMB high checkpoint inhibition
17	Breast	-	-	PIK3CA	c.326A>G, p.Glu109Gly *Probably TMB high	2	5.8	Everolimus, TMB-testing, if TMB high checkpoint inhibition
18	Breast	+	-		not successful			
19	Breast	+	+	MYC	MYC amplification chr8:128748884, CNV 5.47			not actionable
20	Gastric	+	-		no mutation			not actionable
21	PCNSL	+	+	ALK	c.3574C>T, p.Arg1192Trp	23	6.9	TMB-testing, if TMB high checkpoint inhibition.
				ERBB3	c.706T>C, p.Ser236Pro, c.2053C>T,	6	6.9	
				FGFR4	p.Leu685Phe c.1700A>G,	16	7.5	
				JAK3	p.Glu567Gly,	12	6.6	
				MED12	c.3674A>G, p.Lys1225Arg,	26	5.8	
				PDGFRA	c.1975A>G, p.Asn659Asp,	14	35.2	
				RAF1	c.1282A>G, p.Ser428Gly, Probably TMB high	12	10.9	
22	Melanoma	+	+	APC	c.1579A>G, p.Arg527Gly	13	8.2	6 potentially actionable mutations however, several mutation in multiple pathways, indicating resistance. Probably TMB-high, continue current treatment with checkpoint inhibitor.
				BRCA1	c.362A>G, p.Glu121Gly	6	5	
				FGFR1	c.2221T>C, p.Phe741Leu	17	5.6	
				FGFR2	c.1940T>C, p.Leu647Pro	14	20.6	
				MAP2K2	c.193G>A, p.Gly65Ser	2	13.9	
				MET	c.3814A>G, p.Ser1272Gly	19	16.5	
				NRAS	c.35G>A ,p.Gly12Asp	2	5.2	
				SMO	c.1553A>G, p.Glu518Gly Probably TMB high	9	11.9	
23	Breast	+	+	FGFR1	FGFR1 amplification, chr8:38271444, CNV 8.52			Everolimus
				MYC	MYC amplification, chr8:128748884, CNV 8.9			
24	NSCLC	-	+	BRAF	c.1424A>G, p.Lys475Arg c.65A>G,	11	5	Actionable mutation (BRAF), however no change in management as the patient was clinically stable, no LM was detected and cerebral metastasis was controlled after stereotactic radiation. Re-evaluation in case of clinical deterioration
				CDK4	p.Lys22Arg c.172A>G,p.Thr58Ala	2	3.9	
				KRAS		3	4.8	
25	Breast	-	+	EGFR	c.1473A>G, p.Ile491	12	7.3	Actionable mutation (MET, FGFR4), however, because of low allele frequency and clinical deterioration no change in management
				FGFR4	c.2008A>G, p.Ser670Gly	15	6.5	
				MET	c.1120T>C, p.Phe374Leu	2	7.5	
				RET	c.2660A>G, p.Lys887Arg	15	7.7	
26	NSCLC	-	+		not successful			
27	NSCLC	+	+	EGFR	c.2155G>T , p.Gly719Cys	18	17.5	Patient progressed on Afatinib. Best supportive care due to unexpected clinical deterioration.
				EGFR	c.2156G>A p.Gly719Asp	18	3.8	
				EGFR	c.2303G>T, p.Ser768Ile	20	28.5	
				NRAS	c.35G>A, p.Gly12Asp	2	3	
				PTEN	c.1004G>A, p.Arg335Gln	8	5.9	
				TP53	c.527G>T, p.Cys176Phe	5	30.6	

*CSF and/or MRI suggestive of LM; Abbreviations: LM - leptomeningeal metastasis, PM -parenchymal tumor manifestation, AF - allele frequency, NSCLS - non small cell lung cancer, CCC -cholangiocellular carcinoma, CRC - colorectal carcinoma, GBM - glioblastoma, PCNSL - primary CNS lymphoma.
