# Supplementary material for: Therapeutic management of neuro-oncologic patients - potential relevance of CSF liquid biopsy
Source: Theranostics. 2020 Jan 1;10(2):856–66. doi: 10.7150/thno.36884 (PMC6929982; doi:10.7150/thno.36884)
Supplement: Supplementary file 1 — Supplementary figures and tables. [file thnov10p0856s1.pdf]

Supplement:

Supplementary Table 1. Oncomine Focus Assay (OFA) NGS Panel

**Hotspot genes**		**Copy Number Variants**	**Fusion drivers**
**DNA**			**RNA**
AKT1	IDH1	ALK	ALB1
ALK	IDH2	AR	ALK
AR	JAK1	BRAF	AKT3
BRAF	JAK2	CCND1	AXL
CDK4	JAK3	CDK4	BRAF
CTNNB1	KIT	CDK6	EGFR
DDR2	KRAS	EGFR	ERB2
EGFR	MAP2K1	ERB2	ERG
ERB2	MAP2K2	FGFR1	ETV1
ERB3	MET	FGFR	ETV4
ERB4	MTOR	FGFR3	ETV5
ESR1	NRAS	FGFR4	FGFR1
FGFR2	PDGFRA	KIT	GFGR2
FGFR3	PIK3CA	KRAS	FGFR3
GNA11	RAF1	MET	MET
GNAQ	RET	MYC	NTRK1
HRAS	ROS1	MYCN	NTRK2
	SMO	PDGFRA	NRTK3
		PIK3CA	PDGFRA
			PPARG
			RAF1
			RET
			ROS1

Supplementary Table 2: Archer FusionPlex Sarcoma Panel (RNA)

Fusion drivers, splicing or exon skipping variants	
ALK	NCOA2
CAMTA1	NTRK3
CCNB3	PDGFB
CIC	PLAG1
EPC1	ROS1
EWSR1	SS18
FOXO1	STAT6
FUS	TAF15
GLI1	TCF12
HMGA2	TFE3
JAZF1	TFG
MEAF6	USP6
MKL2	YWHAE

Supplementary Table 3: Addendum to Table 4 tumor genetic Mutations without or with unknown significance

#	Gene	Base change	Exon	Reference mRNA	Amino acid change	Allele frequency (%)
**5**	MTOR	c.4418A>G	30	NM_004958.3	p.Asp1473Gly	8.57
	PIK3CA	c.281T>C	2	NM_006218.2	p.Leu94Pro	7.41
	PIK3CA	c.1022C>T	5	NM_006218.2	p.Ala341Val	14.11
	BRAF	c.1522A>G	13	NM_004333.4	p.Thr508Ala	16.73
	FGFR2	c.1924A>G	14	NM_000141.4	p.Ile642Val	33.6
	KRAS	c.430A>G	4	NM_033360.3	p.Thr144Ala	23.45
	ERBB2	c.2006T>C	17	NM_004448.3	p.Val669Ala	6.8
	JAK3	c.1597A>G	12	NM_000215.3	p.Thr533Ala	7.4
**9**	MTOR	c.7273C>T	53	NM_004958.3	p.Pro2425Ser	11.85
	JAK1	c.1921A>G	14(I)	NM_002227.2	p.Met641Val	16.05
	ALK	c.4394T>C	29(I)	NM_004304.4	p.Val1465Ala	13.11
	ALK	c.3608A>G	23(I)	NM_004304.4	p.Asp1203Gly	16.3

	ALK	c.3581T>C	23(I)	NM_004304.4	p.Ile1194Thr	5.3
	ALK	c.3526C>T	23(I)	NM_004304.4	p.His1176Tyr	8.35
	ALK	c.3464T>G	22(I)	NM_004304.4	p.Val1155Gly	36.65
	ALK	c.3395A>G	21(I)	NM_004304.4	p.Glu1132Gly	28.36
	ERBB4	c.2099C>T	18(I)	NM_005235.2	p.Pro700Leu	5.9
	PIK3CA	c.1265T>C	8(I)	NM_006218.2	p.Leu422Ser	12.35
	PIK3CA	c.2078G>A	14(I)	NM_006218.2	p.Arg693His	10.1
	PDGFRA	c.1965G>T	14(I)	NM_006206.4	p.Leu655Phe	9.01
	PDGFRA	c.2488A>G	18(I)	NM_006206.4	p.Lys830Glu	17.15
	KIT	c.1619T>C	10(I)	NM_000222.2	p.Val540Ala	8.3
	KIT	c.1709A>G	11(I)	NM_000222.2	p.Tyr570Cys	12.86
	APC	c.4223A>G	16(I)	NM_000038.5	p.Glu1408Gly	6.55
	EGFR	c.358G>A	3(I)	NM_005228.3	p.Ala120Thr	8.8
	EGFR	c.2170G>A	18	NM_005228.3	p.Gly724Ser	9.9
	SMO	c.1402A>G	8(I)	NM_005631.4	p.Ser468Gly	32.75
	BRAF	c.1692G>A	13(I)	NM_004333.4	p.Met564Ile	11.35
	BRAF	c.1519A>C	13(I)	NM_004333.4	p.Lys507Gln	8.9
	FGFR1	c.695A>C	6(I)	NM_001174067.1	p.His232Pro	17.16
	FGFR1	c.637G>A	6(I)	NM_001174067.1	p.Gly213Arg	17.81
	MYC	c.212T>C	2(I)	NM_002467.4	p.Leu71Pro	7.15
	MYC	c.251T>C	2(I)	NM_002467.4	p.Leu84Pro	7.41
	MYC	c.1151A>G	3(I)	NM_002467.4	p.Glu384Gly	13.25
	RET	c.1838C>T	10(I)	NM_020975.4	p.Pro613Leu	26.5
	FGFR2	c.1022C>T	8(I)	NM_000141.4	p.Thr341Met	9.3
	FGFR2	c.1007A>G	8(I)	NM_000141.4	p.Asp336Gly	8.56
	CCND1	c.367A>G	2(I)	NM_053056.2	p.Lys123Glu	26.21
	KRAS	c.395A>G	4(I)	NM_033360.3	p.Asp132Gly	5.8
	CDK4	c.119C>T	2(I)	NM_000075.3	p.Pro40Leu	9.75
	AKT1	c.85C>T	3(I)	NM_001014431.1	p.Leu29Phe	18.05
	AKT1	c.59A>G	3(I)	NM_001014431.1	p.Lys20Arg	4.9
	ERBB2	c.2291A>G	19(I)	NM_004448.3	p.Asn764Ser	13.7

	ERBB2	c.2308G>A	20(I)	NM_004448.3	p.Glu770Lys	7.35
	ERBB2	c.2345T>C	20(I)	NM_004448.3	p.Val782Ala	6.6
	GNA11	c.523A>G	4(I)	NM_002067.4	p.Thr175Ala	6.35
**16**	PIK3CA	c.994A>G	5	NM_006218.2	p.Ser332Gly	12.06
	ROS1	c.6059A>G	38	NM_002944.2	p.Glu2020Gly	17.05
	EGFR	c.320T>G	3	NM_005228.3	p.Ile107Ser	7.31
	MET	c.1109T>C	2	NM_001127500.1	p.Val370Ala	8.25
	MET	c.3319T>C	16	NM_001127500.1	p.Phe1107Leu	5.27
	MYC	c.1114C>T	3	NM_002467.4	p.Arg372Ter	7.1
	FGFR2	c.944C>T	8(I)	NM_000141.4	p.Ala315Val	10.21
	ERBB3	c.1099G>T	9	NM_001982.3	p.Gly367Cys	7.56
	CDK4	c.904C>T	8	NM_005981.3, NM_000075.3	p.Pro302Ser	32.35
	AKT1	c.79T>C	3(I)	NM_001014431.1	p.Phe27Leu	7.21
	ERBB2	c.2593G>T	21	NM_004448.3	p.Gly865Trp	7.05
**17**	ALK	c.3646A>G	24	NM_004304.4	p.Ser1216Gly	5.7
	RAF1	c.1228G>A	12	NM_002880.3	p.Gly410Arg	16.8
	PIK3CA	c.326A>G	2	NM_006218.2	p.Glu109Gly	5.76
	KIT	c.1591A>G	10	NM_000222.2	p.Ile531Val	10.85
	FGFR4	c.547C>T	5	NM_213647.1	p.Arg183Cys	14.75
	MET	c.2539A>G	11	NM_001127500.1	p.Lys847Glu	10.67
	RET	c.2288A>G	13	NM_020975.4	p.Asn763Ser	5.3
	FGFR2	c.1108A>G	9(I)	NM_000141.4	p.Thr370Ala	11.15
	ERBB3	c.331T>C	3	NM_001982.3	p.Tyr111His	6.4
	CDK4	c.599T>C	5	NM_000075.3	p.Val200Ala	8.8
	MAP2K1	c.385T>C	3	NM_002755.3	p.Phe129Leu	13.4

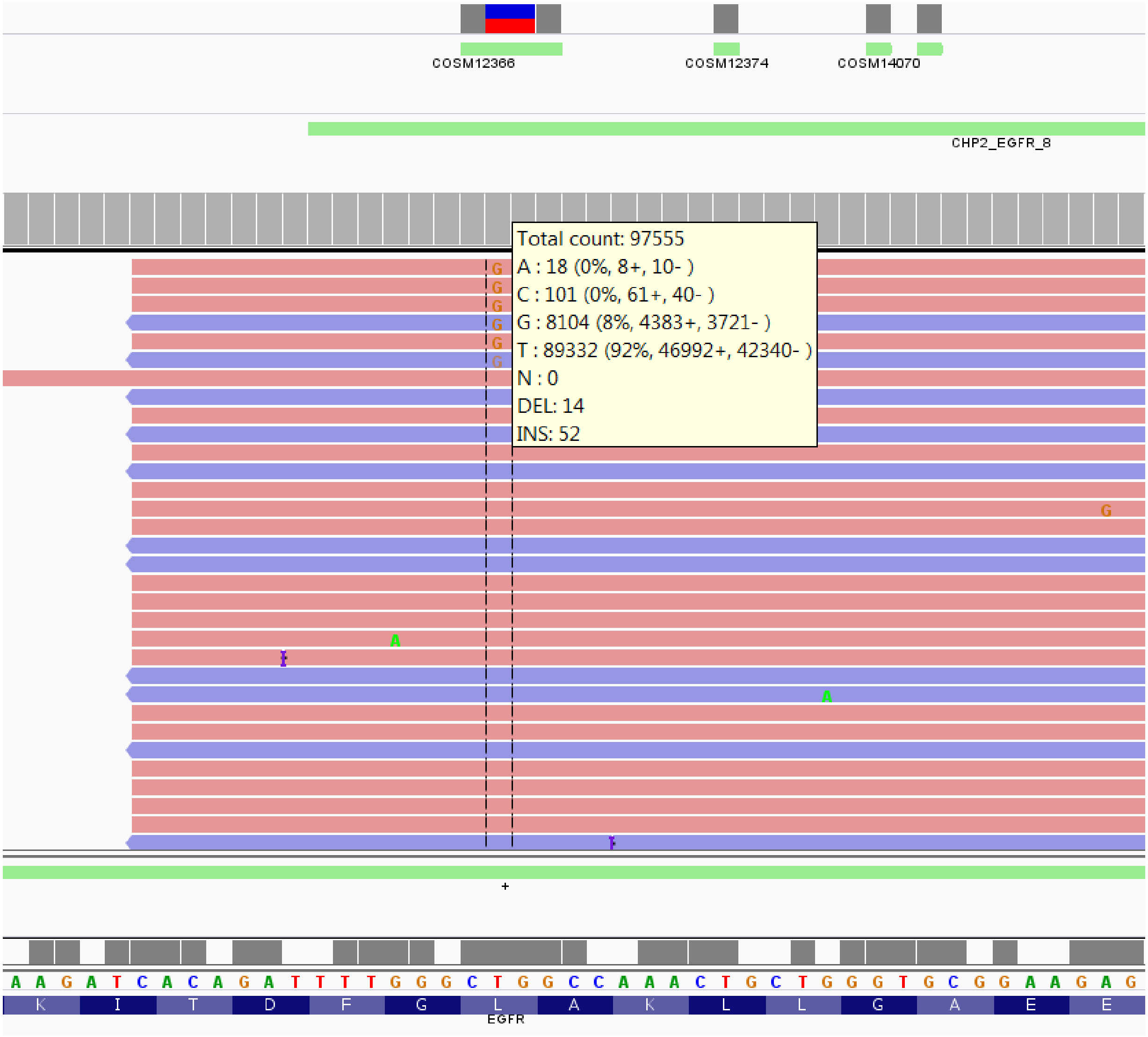

Supplementary Figure 1: Detection of the EGFR L858R mutation in the Integrative Genomics Viewer[1].

The Integrative Genomics Viewer image displays the detection of the EGFR mutation chr7:55259514, c.2573T>G, p.Leu858Arg (L858R) from a liquor biopsy of patient #1 by NGS. The analysis was performed on an Ion Torrent Personal Genome Machine (PGM) with a high average base coverage depth (18674) to achieve an increased sensitivity to detect also low frequency mutations present in liquid biopsies. Sequencing reads were sorted by base. Shown are the total counts as well as counts for each base including the allele frequency and the forward (+, red bars) and reverse (-, blue bars) reads to check for strand read imbalances.

Reference list supplement:

1. Thorvaldsdottir, H., Robinson, J.T., and Mesirov, J.P. Integrative Genomics Viewer (IGV): high-performance genomics data visualization and exploration. Brief Bioinform. 2013; 14: 178–192
